# Supplementary material for: The Impact of the COVID-19 Pandemic on Healthy Lifestyle Behaviors and Perceived Mental and Physical Health of People Living with Non-Communicable Diseases: An International Cross-Sectional Survey
Source: Int J Environ Res Public Health. 2022 Jun 30;19(13):8023. doi: 10.3390/ijerph19138023 (PMC9265593; doi:10.3390/ijerph19138023)
Supplement: Supplementary file 1 [file ijerph-19-08023-s001.zip › ijerph-1739935-supplementary.pdf]

Task Force on Physical Activity for Persons with Disabilities (ISPRM PAPD)

CORONAVIRUS PANDEMIC LIFESTYLE MEDICINE SURVEY

11th July, 2020

[ ] I fully understand what this research project is about, and I consent to participate, all the information I provide can be used by researchers to better understand coronavirus impact on health.

1-DEMOGRAPHICS

1. Gender
   - Male
   - Female
   - Other

2. Age: mm/dd/year

3. Country: _______

4. Education Level
   - Early childhood or Primary
   - Secondary
   - Bachelor or equivalent
   - Master or equivalent
   - Doctoral or equivalent
   - Other: _________

5. Employment Status
   - None
   - Student
   - Employed (part-time or full-time)
   - Unemployed

6. Do you currently have any of the following impairments (you may select more than one):
   - Spinal cord injury
   - Visual impairment or blindness
   - Hearing impairment or deafness
   - Amputation
   - Cerebral palsy
   - Multiple sclerosis
   - Muscular dystrophy

- ALS/PLS
- Short stature
- Other: __________

7. Do you currently have one or more of the following underlying chronic conditions: cardiovascular disease i.e, heart attack and stroke; cancer; chronic respiratory disease i.e. chronic obstructed pulmonary disease and asthma; diabetes
   - Yes, I have one or more of those diseases
   - No, I do not have any of those diseases

2-GENERAL IMPACT OF COVIC PANDEMIC

8. In what ways did the Coronavirus (COVID19) pandemic impact you? (select all that apply)
   - Experienced lockdown (shelter in place)
   - Wore mask in public
   - Maintained social distancing
   - Experienced job loss
   - None of the above
   - Other __________

3-PRE-PANDEMIC HEALTHY LIFESTYLE BEHAVIORS

9. Before the pandemic, how many days per week did you usually get 30 minutes of physical exercise?
   - None
   - 0-1 days/week
   - 2-4 days/week
   - >4 days/week

10. Before the pandemic, how many days per week did you usually eat at least 5 servings of fruits and vegetables?
    - None
    - 0-1 days/week
    - 2-4 days/week
    - >4 days/week

11. Before the Coronavirus pandemic, how many days per week did you usually sleep at least 7-9 hours at night?
    - None
    - 0-1 days/week
    - 2-4 days/week
    - >4 days/week

12. Before the Coronavirus pandemic, on average, for how many uninterrupted hours per day did you sit or recline to watch TV, spend screen time, etc. (this does not include sleeping)?
    - None
    - 1-3 hours
    - 4-7 hours
    - >7 hours

13. Before the pandemic, did you regularly smoke or use tobacco products (cigarettes, cigars, chewing tobacco)?
    - Yes
    - No

4-IMPACT OF THE PANDEMIC ON HEALTHY LIFESTYLE BEHAVIORS

14. During the Coronavirus pandemic, my daily physical exercise was
    - The same as before
    - More than before
    - Less than before
    - Not applicable

15. During the Coronavirus pandemic, my daily consumption of fruits and vegetables has been
    - The same as before
    - More than before
    - Less than before
    - Not applicable

16. During the Coronavirus pandemic, my nightly hours of sleep have been
    - The same as before
    - More than before
    - Less than before
    - Not applicable

17. During the Coronavirus pandemic, my smoking or use of tobacco products has been
    - The same as before
    - More than before
    - Less than before
    - Not applicable

18. During the Coronavirus pandemic, on average, for how many uninterrupted hours per day did you sit or recline to watch T.V., spend screen time, etc. (this *does not* include sleeping)?
    - None
    - 1-3 hours
    - 4-7 hours
    - >7 hours

19. Generally, during the Coronavirus pandemic, I think my physical health and physical fitness has been
    - o The same as before
    - o Better than before
    - o Worse than before
    - o Not applicable

20. Generally, during the Coronavirus pandemic, I think my mental health and emotional wellness (i.e. anxiety, depression, sadness, connectedness) has been
    - o The same as before
    - o Better than before
    - o Worse than before
    - o Not applicable

21. Generally, during the Coronavirus pandemic, I think my daily eating habits have been
    - o The same as before
    - o Better and healthier than before
    - o Worse and less healthy than before
    - o Not applicable

22. During the Coronavirus pandemic, I think I gained
    - o No weight
    - o < 5 lbs
    - o 6-10 lbs
    - o 11-20 lbs
    - o > 20 lbs
    - o I do not know
